# Supplementary material for: Association between chiropractic spinal manipulation for sciatica and opioid-related adverse events: A retrospective cohort study
Source: PLoS One. 2025 Jan 28;20(1):e0317663. doi: 10.1371/journal.pone.0317663 (PMC11774384; doi:10.1371/journal.pone.0317663)
Supplement: S1 Table — (DOCX) [file pone.0317663.s001.docx]

*S1 Table: Sciatica inclusion codes*

| **ICD-10 code** | **Description** |
| --- | --- |
| G54.4 | Lumbosacral root disorders, not elsewhere classified |
| M54.16 | Intervertebral disc disorders with radiculopathy, lumbar region |
| M54.17 | Radiculopathy, lumbosacral region |
| M54.18 | Radiculopathy, sacral and sacrococcygeal region |
| M54.3 | Sciatica |
| M54.4 | Lumbago with sciatica |
| Abbreviations: International Classification of Diseases, 10^th^ Edition (ICD-10) | |
