# Supplementary material for: Association between chiropractic spinal manipulation for sciatica and opioid-related adverse events: A retrospective cohort study
Source: PLoS One. 2025 Jan 28;20(1):e0317663. doi: 10.1371/journal.pone.0317663 (PMC11774384; doi:10.1371/journal.pone.0317663)
Supplement: S2 Table — (DOCX) [file pone.0317663.s002.docx]

*S2 Table: Exclusion criteria for all patients*

| **Code** | **Description** | **Exclusion window (days)** |
| --- | --- | --- |
| Serious pathology (ICD-10) | | |
| C00-C96 | Malignant neoplasm | -365 to 0 |
| F11 | Opioid related disorders (includes heroin) | -365 to 0 |
| F15 | Other stimulant related disorders (includes methamphetamines) |  |
| F14 | Cocaine related disorders | -365 to 0 |
| F15 | Other stimulant-related disorders (includes amphetamines) | -365 to 0 |
| G06 | Intracranial and intraspinal abscess and granuloma | -365 to 0 |
| G83.4 | Cauda equina syndrome | -365 to 0 |
| M46 | Other inflammatory spondyloarthropathies (includes infections) | -365 to 0 |
| M48.4 | Fatigue fracture of vertebra | -365 to 0 |
| M48.5 | Collapsed vertebra, not elsewhere classified | -365 to 0 |
| Z51.5 | Encounter for palliative care | -365 to 0 |
| Alternate conditions (ICD-10) | |  |
| G35 | Multiple sclerosis | -365 to 0 |
| G54.1 | Lumbosacral plexus disorders | -365 to 0 |
| G95 | Other and unspecified diseases of spinal cord | -365 to 0 |
| G99.2 | Myelopathy in diseases classified elsewhere | -365 to 0 |
| M41 | Scoliosis | -365 to 0 |
| M43.16 | Spondylolisthesis, lumbar region | -365 to 0 |
| M43.17 | Spondylolisthesis, lumbosacral region | -365 to 0 |
| M48.0 | Spinal stenosis (includes lumbar stenosis with neurogenic claudication) | -365 to 0 |
| M50.0 | Cervical disc disorder with myelopathy | -365 to 0 |
| M51.0 | Thoracic, thoracolumbar, and lumbosacral intervertebral disc disorders with myelopathy | -365 to 0 |
| M96.1 | Postlaminectomy syndrome, not elsewhere classified | -365 to 0 |
| O00-O9A | Pregnancy | -280 to 365 |
| Z66 | Do not resuscitate | -280 to 365 |
| Z98.1 | Arthrodesis status | -365 to 0 |
| Medications (RxNorm) | | |
| 1819 | Buprenorphine | -365 to 0 |
| 3423 | Hydromorphone | -365 to 0 |
| 4337 | Fentanyl | -365 to 0 |
| 6813 | Methadone | -365 to 0 |
| 56795 | Sufentanil | -365 to 0 |
| Tests | | |
| 3397-7 | Cocaine (presence) in urine | -365 to 0 |
| 3414-0 | Buprenorphine (presence) in urine | -365 to 0 |
| 19550-3 | Methadone (presence) in urine | -365 to 0 |
| 19554-5 | Methamphetamine (presence) in urine | -365 to 0 |
| 59673-4 | Fentanyl (presence) in urine | -365 to 0 |
| Post- surgery | | |
| 1004038 (CPT) | Surgical procedures on the spine (vertebral column) | -365 to 0 |
| 1002796 (CPT) | Anesthesia | -1 to 0 |
| Opioid related adverse drug event (see Table 4) | | -365 to -1 |
| Abbreviations: Central nervous system (CNS); Current Procedural Terminology (CPT); International Classification of Diseases, 10^th^ Edition (ICD-10); normalized names for clinical drugs (RxNorm); custom TriNetX code (NA) | | |
