# Supplementary material for: Association between chiropractic spinal manipulation for sciatica and opioid-related adverse events: A retrospective cohort study
Source: PLoS One. 2025 Jan 28;20(1):e0317663. doi: 10.1371/journal.pone.0317663 (PMC11774384; doi:10.1371/journal.pone.0317663)
Supplement: S3 Table — (DOCX) [file pone.0317663.s003.docx]

S3 Table: Variables controlled for in propensity score matching

| **Variable/Code** | **Description** |
| --- | --- |
| Demographics | Patient age, sex, race, and ethnicity |
| Diagnoses (ICD-10) | |
| E08-E13 | Diabetes mellitus |
| F10-F19 | Mental and behavioral disorders due to psychoactive substance use, including alcohol, opioid, and other substances |
| F10 | Alcohol related disorders |
| F13 | Sedative, hypnotic, or anxiolytic related disorders |
| F17 | Nicotine dependence (includes smoking, vaping) |
| F30-F39 | Mood disorders (includes depression, bipolar) |
| I10-I1A | Hypertensive diseases |
| J44.9 | Chronic obstructive pulmonary disease, unspecified |
| K70-K77 | Diseases of liver |
| M19 | Other and unspecified osteoarthritis |
| N18 | Chronic kidney disease |
| Prescription medications | |
| 7242 (RxNorm) | Naloxone |
| 25480 (RxNorm) | Gabapentin |
| AD100 (VANDF) | Alcohol deterrents |
| CN101 (VANDF) | Opioid analgesics |
| CN300 (VANDF) | Sedatives/hypnotics (includes benzodiazepines) |
| MS200 (VANDF) | Skeletal muscle relaxants |
| VA000 (VANDF) | Medications (any) |
| Social determinants of health (ICD-10) | |
| Z55-65 | Adverse socioeconomic and psychosocial circumstances |
| Procedures (CPT) | |
| 1003143 | Surgery |
| 1002796 | Anesthesia |
| Abbreviations: Anatomical Therapeutic Chemical Classification (ATC), Current Procedural Terminology (CPT), normalized names for clinical drugs (RxNorm); International Classification of Diseases, 10^th^ Edition (ICD-10); Veterans Health Administration National Drug File (VANDF) | |
