# Supplementary material for: Association between chiropractic spinal manipulation for sciatica and opioid-related adverse events: A retrospective cohort study
Source: PLoS One. 2025 Jan 28;20(1):e0317663. doi: 10.1371/journal.pone.0317663 (PMC11774384; doi:10.1371/journal.pone.0317663)
Supplement: S4 Table — (DOCX) [file pone.0317663.s004.docx]

S4 Table: Opioid-related adverse drug events

| **Variable/Code** | **Description** |
| --- | --- |
| HZ85ZZZ (ICD-10-PCS) | Medication management for substance abuse treatment, naloxone |
| HZ95ZZZ (ICD-10-PCS) | Pharmacotherapy for substance abuse treatment, naloxone |
| J2310 (HCPCS) | Injection, naloxone hydrochloride, per 1 mg |
| J2315 (HCPCS) | Injection, naloxone, depot form, 1mg |
| T40.0 (ICD-10) | Poisoning by, adverse effect of and underdosing of opium |
| T40.2 (ICD-10) | Poisoning by, adverse effect of and underdosing of other opioids |
| T40.3 (ICD-10) | Poisoning by, adverse effect of and underdosing of methadone |
| T40.4 (ICD-10) | Poisoning by, adverse effect of and underdosing of other synthetic narcotics |
| Abbreviations: Normalized names for clinical drugs (RxNorm); Healthcare Common Procedure Coding System (HCPCS); International Classification of Diseases, 10^th^ Edition (ICD-10); ICD-10 Procedure Coding System (ICD-10-PCS) | |
